# Impact of DNA damage repair alterations on prostate cancer progression and metastasis

**DOI:** 10.3389/fonc.2023.1162644

**Published:** 2023-06-26

**Authors:** Natalia Lukashchuk, Alan Barnicle, Carrie A. Adelman, Joshua Armenia, Jinyu Kang, J. Carl Barrett, Elizabeth A. Harrington

**Affiliations:** ^1^ Translational Medicine, Oncology Research and Development (R&D), AstraZeneca, Cambridge, United Kingdom; ^2^ Oncology Data Science, Oncology Research and Development (R&D), AstraZeneca, Cambridge, United Kingdom; ^3^ Global Medicines Development, Oncology Research and Development (R&D), AstraZeneca, Gaithersburg, MD, United States; ^4^ Translational Medicine, Oncology Research and Development (R&D), AstraZeneca, Waltham, MA, United States

**Keywords:** DNA damage response, homologous recombination repair, alterations, prognosis, prostate cancer

## Abstract

Prostate cancer is among the most common diseases worldwide. Despite recent progress with treatments, patients with advanced prostate cancer have poor outcomes and there is a high unmet need in this population. Understanding molecular determinants underlying prostate cancer and the aggressive phenotype of disease can help with design of better clinical trials and improve treatments for these patients. One of the pathways often altered in advanced prostate cancer is DNA damage response (DDR), including alterations in *BRCA1/2* and other homologous recombination repair (HRR) genes. Alterations in the DDR pathway are particularly prevalent in metastatic prostate cancer. In this review, we summarise the prevalence of DDR alterations in primary and advanced prostate cancer and discuss the impact of alterations in the DDR pathway on aggressive disease phenotype, prognosis and the association of germline pathogenic^1^ alterations in DDR genes with risk of developing prostate cancer.

## Introduction: prostate cancer and treatment landscape

Prostate cancer is the third most common cancer worldwide, with 1,414,259 new cases and 375,304 deaths in 2020, and the 5^th^ leading cause of cancer deaths worldwide (1, 2). Approximately 80% of men with prostate cancer are diagnosed with localised prostate cancer, and their 10-year survival is up to 99% if diagnosed early. Approximately 10–20% of men with advanced prostate cancer will develop castration-resistant prostate cancer (CRPC) within five years, and at least 84% of these men will have metastases at the time of CRPC diagnosis. Men with metastatic CRPC have poor outcomes (3–5).

Metastatic prostate cancer is a broad term used to describe a range of advanced disease states that are no longer organ-confined. This group includes *de novo* metastatic castration-sensitive prostate cancer (mCSPC), as well as cancers that progress during or after androgen deprivation therapy (ADT), termed metastatic castration-resistant prostate cancer (mCRPC) (6). Based on clinical trials, median overall survival (OS) for patients with mCRPC is approximately three years, and is even less in the real-world setting (7). Approximately half of patients with mCRPC may receive only one line of active treatment, with diminishing benefit observed with the use of subsequent therapies (5, 8–11).

**Figure 1:**
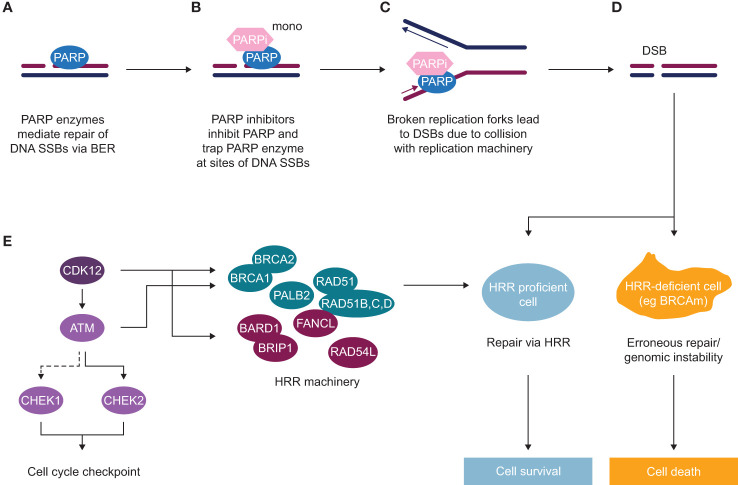
Mechanism of action of PARP inhibitors (as monotherapy) in HRR deficient background **(A)** Poly(ADP-ribose) polymerase (PARP) enzymes are recruited to sites of DNA single-strand breaks (SSBs) and mediate their repair *via* base excision repair (BER). **(B)** PARP inhibitors inhibit PARP and trap PARP enzyme at sites of SSBs. **(C)** In replicating cells, due to collision of trapped PARP with replication machinery, broken replication forks occur, which lead to double-strand breaks (DSBs). **(D)** In normal cells or HRR proficient cells, such DSBs get repaired *via* an accurate DNA repair process of HRR, with some of the key HRR factors depicted in panel **(E)** In HRR-deficient cells, such as BRCA mutant cells or with alteration in another HRR gene, lack of efficient HRR repair leads to erroneous DNA repair and subsequent genomic instability and cell death. **(E)** HRR is an accurate DNA repair pathway, which operates in S/G2 cells where the sister chromatid is present. HRR factors include DNA damage sensors and factors involved in direct repair [BRCA1, BRCA2, PALB2, RAD51 and RAD51 paralogues **(B–D)**], as well as DNA repair by either interacting with key HRR factors (BARD1, BRIP1, FANCL, RAD54L) or regulating HRR gene expression (CDK12), as well as key kinases inducing cell cycle arrest to allow repair to occur (ATM, CHEK1, CHEK2).

A variety of life-prolonging agents are approved for the mCRPC population overall, however the most used medicines for patients with mCRPC remain mainly chemotherapy (docetaxel/cabazitaxel) and new hormonal agents (NHAs; e.g abiraterone and enzalutamide) (8, 12–14). Once patients with mCRPC have failed NHA, the benefit from approved therapeutic options appears substantially diminished (12, 15–22). As such, there is a high unmet medical need for patients who have progressed after NHA treatment, and many efforts to find more effective treatment options have failed in the past decade. Recently, poly(ADP-ribose) polymerase inhibitors (PARPi) treatment as monotherapy demonstrated radiographic progression-free survival (rPFS) and OS improvement in biomarker-selected patient populations with an underlying alteration in the homologous recombination repair (HRR) pathway (HRRm, or *BRCA1/2* alterations) (23–29) and have been approved as monotherapy for patients with HRR mutant (HRRm) mCRPC post NHA (25, 30, 31). The mechanism of action of PARP inhibitors as monotherapy is described in **Figure 1**. The androgen receptor, in addition to its role in binding androgen and stimulating prostate cancer cell growth (32), also contributes towards the general repair of DNA damage, including damage not normally repaired by HRR (33–35). This demonstrates that androgen receptor and PARP are both important for the repair of DNA damage in prostate cancer cells and highlights the importance of crosstalk (interaction) between both mechanisms. This evidence provided the rationale for the clinical development of co-administration of NHAs and PARP inhibitors (36). The clinical benefit of PARPi in combination with NHA has been demonstrated in biomarker-selected and biomarker-unselected populations in first-line mCRPC (**Table 1**) (36–39) and approvals have been granted in some regions (40).

**Table 1:** Clinical trials involving PARP inhibitors in mCRPC evaluating the predictive value of BRCA mutation/HRR mutation/HRD status.

Study	Phase	Treatments	Target population	DDRm prospective selection	Primary endpoint	Findings	Reference
PARPi (single agent)							
**PROfound**	**III**	Two arms (2:1): Olaparib 300 mg BID vs physician’s choice of enzalutamide or abiraterone	mCRPC post NHA	Yes	rPFS in Cohort A (*BRCA1/2*m and/or *ATM*m) by BICR	HR 0.34 (95% CI 0.25–0.47), median 7.4 vs 3.6 months	(25)
**TRITON-3**	**III**	Two arms (2:1): Rucaparib 600 mg BID vs physician’s choice of docetaxel, enzalutamide or abiraterone	mCRPC post NHA	Yes	rPFS by BICR	BRCA subgroup: HR 0.50 (95% CI 0.36–0.69), 11.2 vs 6.4 months; in the total population: HR 0.61; (95% CI 0.47–0.80), median 10.2 vs 6.4 months	(28)
**TALAPRO-1**	**II**	Single arm: Talazoparib 1 mg/day	mCRPC post 1–2 cycles TBC and NHA	Yes	Best ORR (RECIST)	ORR: 29.8% (95% CI 21.2–39.6)	(26)
**TRITON-2**	**II**	Single arm: Rucaparib 600 mg BID	mCRPC post 1 cycle TBC and NHA	Yes	Confirmed ORR (RECIST)	ORR: 43.5% (95% CI 31.0–56.7)	(29)
**GALAHAD**	**II**	Single arm: Niraparib 300 mg/day	mCRPC post 1 cycle TBC and NHA	Yes	Confirmed ORR in BRCAm (RECIST)	ORR: 34.2% (95% CI 23.7–46.0)	(27)
**TOPARP-A**	**II**	Single arm: Olaparib 400 mg BID	mCRPC post docetaxel	No	Composite RR (PSA + RECIST + CTC)	ORR in HRRm: 33% (95% CI 20–48)	(23)
**TOPARP-B**	**II**	Two arms (1:1): Olaparib 400 mg BID vs olaparib 300 mg BID	mCRPC post TBC	Yes	Composite RR (PSA + RECIST + CTC)	400 mg: ORR 54% (95% CI 39–69); 300 mg: 37% (95% CI 23–53)	(24)
PARPi + NHA combinations							
**PROpel**	**III**	Two arms (1:1): Olaparib 300 mg BID + abiraterone (1000 mg) + prednisone or prednisolone (5 mg) once daily vs placebo + abiraterone (1000 mg) + prednisone or prednisolone (5 mg) once daily	1L mCRPC (no prior chemo or NHA in metastatic setting)	No	rPFS by investigator	HR: 0.66 (95% CI 0.54–0.81), median 24.8 vs 16.6 months	(37)
**Magnitude**	**III**	Two arms (1:1): Niraparib 200 mg + abiraterone (1000 mg) + prednisone or prednisolone (10 mg) once daily versus placebo + abiraterone (1000 mg) + prednisone or prednisolone (10 mg) once daily	1L mCRPC (no prior chemo or <4 months prior NHA in metastatic setting)	Yes	rPFS in patients with HRRm, by BICR	HR: 0.73 (95% CI 0.56–0.96), median 16.5 vs 13.7 months	(38)
**TALAPRO-2**	**III**	Two arms (1:1): Talazoparib 0.5 mg/day + enzalutamide 160 mg once daily vs placebo + enzalutamide 160 mg once daily	1L mCRPC	No	rPFS by BICR	In ITT (all comers) HR: 0.63 (95% CI 0.51–0.78), median NR vs 21.9 months	(39)
**Study 8**	**II**	Two arms (1:1): Olaparib 300 mg BID + abiraterone (1000 mg) + prednisone or prednisolone (5 mg) versus once daily placebo + abiraterone (1000 mg) + prednisone or prednisolone (5 mg) once daily	mCRPC post docetaxel and no prior NHA in metastatic setting	No	rPFS by investigator	HR: 0.65 (95% CI 0.44–0.97), median 13.8 vs 8.2 months	(36)

1L, first line; BICR, blinded independent central review; BID, twice daily; BRCA1/2m, mutations in BRCA1 and BRCA2; CI, confidence interval; CTC, Circulating Tumour Cells; DDRm, DNA Damage Repair gene mutations; HR, hazard ratio; HRRm, homologous recombination repair gene mutations; mCRPC, metastatic castration-resistant prostate cancer; NHA, new hormonal agent; NR, not reached; ORR, objective response rate; PARPi, poly(ADP-ribose) polymerase inhibitors; PSA, prostate-specific antigen; rPFS, radiological progression-free survival; RR, response rate; TBC, Taxane Based Chemotherapy.

Despite recent progress in treatments for metastatic disease, there is a high unmet need in this patient population (5, 41). Understanding the biology underlying primary and metastatic prostate cancer, and the differences in prognosis, can help improve prostate cancer treatment and patient outcomes.

### Prostate cancer genomic landscape

Comprehensive molecular characterisation through genetic profiling has revealed a complex and heterogenous genomic landscape of prostate cancer. Multiple landmark genomics studies have identified some of the most recurrent altered genes and pathways in advanced prostate cancer, including genes involved in androgen signalling (50%), PI3K signalling (40%), the cell cycle (24%), WNT/beta-catenin signalling (19%), as well as genes involved in DNA damage response (DDR; 27%), with significant enrichment of all pathways observed in mCRPC (42–46). Alterations in the RAS pathway, including hotspots in *BRAF* or deleterious alterations in *NF1* or *RASA1* are detected at lower prevalence than other genes, at around 8% (43). These studies have also highlighted the presence of distinct genomic subtypes defined by rearrangements involving the *ERG* transcription factor (46%), or hotspot mutations in *SPOP* (8–11%) and *FOXA1* (3%) (44). Due to the highly complex nature of this disease, patients with prostate cancer could greatly benefit from better means of molecular stratification to better select appropriate anti-cancer therapies.

### Prevalence of alterations in the HRR/DDR pathway in prostate cancer

Here we discuss alterations in the DDR pathway, which are frequent in prostate cancer and particularly in advanced stages of disease. DDR is a tightly coordinated pathway that enables cells to control and regulate DNA damage that arises every day. Accumulating damage can lead to mutations and promote genomic instability, which is one of the hallmarks of cancer development (47, 48). Alterations in DDR genes are found to be frequently mutated in many types of cancer, including prostate cancer, where around 23–31% of patients with advanced prostate cancer have been reported to have alterations in DDR genes (43, 45, 46, 49).

As reviewed recently by Morgado & Mateo (50), DDR mutant cancer has been a term used broadly to describe genomic alterations in any gene involved in DDR, including HRR or mismatch repair (MMR) alterations, which have different implications and therapeutic targets in primary and metastatic prostate cancer. Within DDR genes, alterations in genes involved in HRR are most prevalent (23–28%) in mCRPC, with alterations in other pathways of DDR, such as MMR (3–4%) or Fanconi anaemia (FA; around 5%), found at lower prevalence (42, 43, 49, 51, 52).

DNA repair is a complex process that involves sensing DNA damage and downstream signalling cascades to promote DNA repair by recruiting DDR factors and triggering cell cycle checkpoints to allow cells to repair DNA (53, 54). Cancer cells often deregulate the DDR pathway *via* genomic alterations or epigenetic silencing of DDR genes, which can lead to genomic instability – one of the hallmarks of tumourigenesis (47). Alterations in some of the DDR genes, particularly genes involved in HRR in prostate cancer, are associated with worse prognosis and a higher likelihood of developing metastatic disease (55). The HRR pathway is an accurate pathway that regulates the repair of DNA damage, such as double-strand breaks (DSBs). This pathway relies on the presence of sister chromatid, and therefore only operates in S and G2 stages of the cell cycle when the homologous chromatid is available, whereas the NHEJ pathway takes place in all stages of the cell cycle or in quiescent cells. The HRR pathway is also required for repair of DSBs arising during inter-strand crosslink repair, a process that includes FA factors and other pathways such as nucleotide excision repair and translesion synthesis (47, 48, 56). There are multiple DDR factors that have a direct and indirect role in the HRR pathway: DNA damage sensors (i.e. MRN complex and ATM), signal mediator proteins (i.e. BARD1, BRCA1, PALB2, BRCA2, FANCL, RAD54L), effector proteins directly involved in DNA repair (i.e. RAD51, RAD54L) *via* strand invasion and replication fork stabilisation, downstream signalling to trigger cell cycle checkpoints (ATM, CHEK2, CHEK1) or regulating transcription of HRR genes (i.e. CDK12); (**Figure 1E**) (57, 58).

Multiple genes in the HRR pathway are altered in several types of cancers, including in prostate cancer; these include: *BRCA1*, *BRCA2*, *ATM*, *CDK12*, *PALB2*, *BRIP1*, *CHEK1*, *CHEK2*, *RAD51B*, *RAD51C*, *RAD51D*, *RAD54L, BARD1* and *FANCL* (**Table 2**).

**Table 2:** Prevalence of HRRm in primary and metastatic prostate cancer.

Gene	Prevalence range primary PC (%)	Prevalence range in mCRPC (%)	References
*BRCA1/BRCA2*	2.5–6.5	11–13	(23, 43, 45, 46, 49, 51)
*ATM*	0.5–3	4–6	
*BRCA1/2/ATM*	6–7	13–19	
*CDK12*	2–3	1.3–8	
*CHEK2*	0–1	1.4–2	
*PALB2*	0–1	0.3–3	
*RAD51B*	0	0–0.7	
*RAD51C*	0–0.9	0–1.8	
*RAD51D*	0–0.6	0–0.6	
*RAD54L*	0	0–0.6	
*BRIP1*	0–0.3	0–0.3	
*CHEK1*	0–1	0.9–2	
*BARD1*	0–0.4	1.2–1.4	
*FANCL*	0–0.7	1.2	
*HRRm*	10–11	23–28	

Pathogenic/likely pathogenic or deleterious/likely deleterious alterations were included, and variants of uncertain significance were excluded where possible. Data from available public sources may differ in the determination of pathogenicity of mutations. Some of the datasets (42, 43, 59) have been reanalysed by AstraZeneca using cBioPortal (60). Co-occurring HRR gene mutations were excluded from analysis of total HRRm prevalence where possible. Some of the germline variants were excluded in MSK-IMPACT and MSK-DFCI datasets (43, 59) due to patients consent.

HRRm, homologous recombination repair gene mutations; mCRPC, metastatic castration-resistant prostate cancer; PC, prostate cancer.

The most well characterised genes involved in HRR are tumour suppressor genes *BRCA1* and *BRCA2* (also referred to as ‘BRCA’ genes), alterations in which have been associated with prostate cancer as well as breast, ovarian and pancreatic cancer (61). BRCA1 and BRCA2 proteins play a key role in HRR, where BRCA1 plays a role in the early step of determining DSB repair pathway choice to promote resection and channel it to HRR, for which interaction with BARD1 is important. Later, BRCA1 interacts with PALB2 to bring BRCA2, which promotes RAD51 filament formation and strand invasion, a key step in HRR (62, 63). RAD54L plays a supportive role by promoting RAD51 filament stabilisation (58) BARD1 and BRIP1 are important BRCA1 interacting partners, promoting repair pathway choice, DNA repair and DDR checkpoints (64, 65). There are other important HRR genes that play a direct or indirect role in HRR, such as a kinase, ATM, which is involved in response to DSBs by phosphorylating key DDR proteins to propagate signalling to promote repair or to arrest the cell cycle. ATM directs repair of DSBs associated with replication to HRR by promoting efficient DNA resection (66). One of the key downstream targets of ATM is CHEK2 kinase, the phosphorylation of which leads to activation of the G1 checkpoint (67–70). On the other hand, CHEK1 kinase is phosphorylated by ATR and is involved in triggering G2/M as well as intra-S checkpoints (68, 69, 71–73). CDK12, a kinase with an indirect role in HRR, regulates transcription of HRR genes (74, 75). Alterations in CDK12 lead to a unique tandem duplication genotype (76).

In prostate cancer, alterations in genes involved in HRR are enriched in later/advanced stages of disease compared with primary prostate cancer (46, 55).

Around 10−11% of patients with primary cancer harbour HRR alterations, whereas between 23% and 28% of patients with mCRPCs have loss-of-function mutations in genes involved in the HRR pathway of DDR in the tumour (**Table 2**).

In the PROfound clinical trial (a randomised, open-label, Phase III trial evaluating the PARP inhibitor olaparib in men with mCRPC who had disease progression while receiving a new hormonal agent, e.g. enzalutamide or abiraterone), an alteration in one or more of 15^2^ prespecified HRR genes was detected in 28% of over 4000 patients screened (25). Very similar prevalence of HRRm was also observed in the Phase III PROpel trial (double-blind, Phase III trial of abiraterone and olaparib versus abiraterone and placebo in patients with mCRPC in the first-line setting), where patients were enrolled regardless of HRR status; 28% of patients enrolled had alterations in HRR genes (37). Comparable prevalence of HRR/DDR genomic alterations were also observed in other clinical trials and datasets involving advanced prostate cancer tumours. In the TOPARP-A clinical trial (Phase II trial in which patients with mCRPC were treated with olaparib), 33% of patients harboured genomic alterations specific to DDR genes (23). Chung et al. used real-world data from routine prospective genomic profiling and showed that 23% of patients had alterations in genes involved in the HRR pathway (49) and a similar prevalence of HRR gene mutations was also demonstrated by Abida et al. in 429 patients with mCRPC (42).

Mutations in the BRCA genes (*BRCA1* and/or *BRCA2*) are the most prevalent HRR gene mutations in mCRPC (with *BRCA2* more prevalent than *BRCA1*), with *ATM* being the second most frequently mutated (23, 43, 46, 49). In mCRPC, prevalence of BRCAm ranges from around 11% to 13%, and *ATMm* from 4% to 6% (**Table 2**). The next most prevalent mutations in HRR genes in mCRPC are *CDK12* (1.3–8%), *CHEK2* (1.4–2%), *PALB2* (0.3–3%), and *CHEK1* (0.9–2%) (**Table 2**). The prevalence of alterations in other HRR genes in prostate cancer is low and ranges between 0% and 1.8% (**Table 2**).

In summary, between around 23–28% of patients have deleterious alterations in HRR/DDR genes in metastatic prostate cancer, with most studies reporting alterations in the tumour (**Table 2**) (25, 37, 42, 49)

The origin of alterations in DDR genes found in tumour can be either germline or somatic. The relative ratio of pathogenic germline to somatic mutation events in DDR is highly dependent on the HRR genes interrogated. Lai et al. (52) identified that the ratio of germline to somatic alterations in *BRCA1*/2 genes was roughly 1:1, whereas other HRR genes such as *ATM* and *CDK12* had a much higher ratio of HRRm that were somatic in origin relative to germline with 70% of *ATM* alterations and 89% of *CDK12* alterations being somatic, based on a validated computational algorithm (77). The prevalence of germline alterations in HRR genes in mCRPC is 12% (78), with 6% in *BRCA1/2*, *ATM* 1.6%, *CHEK2* 1.9% and other HRR genes below 1%. In primary prostate cancer the overall prevalence of germline DDR alterations is lower at 4.6%, with highest prevalence of 1% in *ATM*, and 0.6% in *BRCA1* and 0.2% in *BRCA2* (78).

Loss of function of both alleles is needed for inactivation of HRR gene function. Biallelic loss-of-function rate of HRR gene mutations in prostate cancer is high at 73%, with the highest rate in *BRCA2* (>90%) and *ATM* (around 75%); beyond these, the rate is variable (ranging between under 10% for *BRIP1* and over 60% for *CDK12)* (52), suggesting alterations in HRR genes are important drivers of tumourigenesis for prostate cancer. Biallelic inactivation rate was high for both germline and somatic BRCA alterations (52, 79), suggesting both play an important role in prostate cancer tumourigenesis.

Beyond HRR gene alterations described above, epigenetic silencing of another HRR gene – *XRCC3* – has been reported in prostate adenocarcinoma (80). *XRCC3* is one of the RAD51 paralogues and its role in HRR has been previously reported (81). Interestingly, *XRCC3* alterations were mutually exclusive with alterations in BRCA genes in a pan-cancer dataset, similar to other HRR genes, which confirms its functional importance in the HRR pathway. Depletion of this gene sensitised cells to PARP inhibition preclinically (80), which warrants further investigation of loss of *XRCC3* expression as a potential biomarker for PARPi sensitivity in clinic.

### Genomic instability/HRD in prostate cancer

A consequence of deficiency in the HRR pathway (HRD) is the accumulation of DNA damage leading to genomic instability signatures, or scars, over time. In addition to alterations in HRR genes, HRD/genomic instability can be another way to identify patients who might benefit from PARP inhibitors. HRD has been associated with a clinical benefit for PARP inhibitor treatment in ovarian cancer (82–85) and to platinum-based chemotherapy in breast cancer (86); however, there is lack of evidence of clinical utility of HRD in other tumour types.

In prostate cancer, genomic instability/HRD (as measured by genome-wide loss of heterozygosity [gLOH]) is generally lower than in ovarian, breast or pancreatic cancers (52, 79). Biallelic alterations in BRCA genes or HRR genes are associated with higher gLOH/HRD scores compared with BRCA wild type (wt) or HRR wt prostate cancer tumours, respectively, which is consistent with these alterations leading to deficiency in the HRR pathway (52, 79). However, there are no data on the clinical utility of gLOH/HRD scores in prostate cancer. The distinction between HRD-positive and HRD-negative tumours, based on genomic instability in prostate cancer, is not clear relative to ovarian and breast cancer. The cut-off to identify HRD-positive tumours in prostate cancer would need to be robustly defined, and this is likely to be more challenging than in ovarian cancer due to a lower dynamic range in prostate cancer. Interestingly, Zurita et al. assessed the relationship between genome instability and clinical features and identified that higher genomic instability was associated with higher risk of disease progression to CRPC (87). Recently, a functional biomarker of HRR has been developed based on measuring nuclear foci formed by the key HRR factor, RAD51, at DNA damage sites, which is currently being implemented in clinical trials (88).

Beyond HRR, alterations in other genes in the DDR pathway are relatively low, however some are potentially actionable. Deficiency in MMR is the underlying cause of the microsatellite instability-high (MSI-H) phenotype, which is a biomarker of response to immune checkpoint blockade therapy (89). Prevalence of alterations in the MMR pathway (*MLH1, MSH2, MSH6, PMS2*) or MSI-H in prostate cancer is much lower than HRRm, at around 3–5% (49). Interestingly, mutual exclusivity between genomic instability/HRD and MSI-H has been reported across tumours (79, 90). In prostate cancer, 12.8% of *BRCA1* and 3.4% of *BRCA2* alterations co-occurred with MSI-H, and 46.3% of MSI-H had at least one HRR gene mutation; however, most *BRCA* mutations in the MSI-H segment were monoallelic (90, 91).

### Increased prevalence of HRR alterations in metastatic vs primary/early prostate cancer

Enrichment of *BRCA1/2*, *ATM* and *CDK12* mutations in advanced prostate cancer has been documented in the literature by several studies (43, 45, 49, 92). Armenia et al. performed a large study that analysed 680 primary tumours and 333 metastatic samples and identified HRR defects in 10% and 27% of the primary and metastatic samples, respectively (43). Similarly, Abida et al. (45) observed an increase in HRR alterations according to disease progression, with 10% of HRR alterations detected in primary tumours, 14% in castration-sensitive prostate cancer and 27% in CRPC. The high representation of *BRCA2* mutations in advanced/metastatic prostate cancer is considered to be a consequence of *BRCA2* mutations being associated with a particularly aggressive phenotype (49, 93–95) rather than these mutations (e.g. androgen receptor mutations and amplifications) being acquired under treatment with standard therapies (96). An increase in the prevalence of DDR alterations in metastatic compared with primary prostate cancer could either be due to disease progression or therapy exposure, or could be due to a worse prognosis for patients with mutations/alterations in DDR genes (DDRm) prostate cancer. Recent analysis of paired tumour samples from patients with prostate cancer (treatment-naïve and metastatic tumour samples) showed that in most cases, alterations in DDR genes were already present in the primary prostate cancer sample, suggesting that this is an early event in tumourigenesis (97). On the contrary, alterations of *AR*/*TP53*/*RB1* are enriched at later disease stages (97). These data suggest that genomic instability associated with alterations in HRR genes leads to a more aggressive disease, which is more likely to metastasise, highlighting the need to treat those patients early.

### Increased prostate cancer risk for germline alterations in DDR genes

Family history is an important risk factor to be considered for development of prostate cancer. Germline alterations in MMR genes (*MLH1, MSH2, MSH6, PMS2*) and the HRR pathway (*BRCA1/2, ATM, PALB2, CHEK2*), particularly *BRCA* and *ATM*, are associated with increased risk of hereditary prostate cancer, as reported by National Comprehensive Cancer Network (NCCN) guidelines (98). The proportion of patients with prostate cancer with germline mutations in DDR genes increases from around 5% in primary cancer to 12–16% in mCRPC, indicating a more aggressive nature of disease with germline DDR alterations (46, 78, 99).

Men with germline pathogenic *BRCA1/2* mutations have an increased risk of prostate cancer and the relative increase in risk of prostate cancer in men <65 years ranges from 1.8-fold to 3.8-fold for germline *BRCA1m* carriers (100, 101) and from 2.5-fold to 8.6-fold for germline *BRCA2m* carriers (102–105) compared with non-carriers. A large meta-analysis of 8 cohort, 7 case control, 4 case series, 28 frequency and 11 survival studies found that being a *BRCA* mutation carrier (*BRCA1* and/or *BRCA2*) was associated with a significant increase in prostate cancer risk (odds ratio [OR] =1.90; 95% CI 1.58, 2.29), with *BRCA2* mutations being associated with a greater risk of prostate cancer than *BRCA1* (106) (**Table 3**). Lifetime risk of cancer ranged between 19–61% for *BRCA2* carriers, and 7–26% for *BRCA1* carriers (108). A prospective study, IMPACT, which is evaluating targeted screening using prostate-specific antigen (PSA) in men with germline pathogenic *BRCA1/2* mutations, has reported a higher incidence of prostate cancer in *BRCA2* carriers compared with controls at interim analysis (114). Furthermore, germline pathogenic *BRCA2* mutations are associated with a particularly aggressive phenotype and poor outcomes (93, 94). Germline pathogenic mutation status of *BRCA* and *ATM* distinguishes risk for lethal and indolent prostate cancer and is associated with earlier age at death and shorter survival time (115). Germline pathogenic variants in *ATM* lead to an approximate fourfold elevated risk of developing prostate cancer, and in addition, they are more likely to develop the disease earlier (109, 116). Beyond *BRCA* genes, there is evidence of elevated prostate cancer risk for *CHEK2* heterozygotes (110, 111) and increased incidence of germline pathogenic *CHEK2*, *BRIP1* and *PALB2* mutations in familial cases of prostate cancer (**Table 3**) (64, 112, 117).

**Table 3:** Hereditary cancer risk for HRR genes.

Gene name	Prostate cancer risk	
	RR or OR (95% CI)	References
*BRCA1*	RR 1.8–3.8	(101, 108, 100)
	OR 1.35 (1.03–1.76) OR 1.83 (0.35–9.51)	(106, 107)
*BRCA2*	RR 2.5–8.6	(102–105, 108)
	OR 2.64 (2.03–3.47) OR 3.92 (1.34–11.47)	(106, 107)
*ATM*	OR 4.4 (2.0–9.5) OR 3.83 (1.09–13.41)	(107, 109)
*CHEK2*	Higher risk suggested (OR 1.837) increased incidence of *CHEK2* mutations in family cases compared with unselected cases and the general population OR 0.47 (0.15–1.45)	(107, 110, 111)
*PALB2*	Family segregation case	(112)
*BRIP1*	Increased prevalence in familial and young-onset cases compared with controls OR 2.4 (0.25, 23.4)	(105, 113)
*RAD51C*	Unknown	
*RAD51D*	Unknown	
*BARD1*	OR 0.32 (0.03–3.55)	(107)
*CDK12^a^, CHEK1, RAD54L, RAD51B, FANCL^b^*	Unknown	

^a^CDK12, not likely to be associated as very few/no germline mutations; most are somatic; ^b^FANCL, associated with cancer predisposition syndrome Fanconi anaemia. CI, confidence interval; HRR, homologous recombination repair; OR, odds ratio; RR, relative risk, SRR, standardised relative risk.

In addition to prostate cancer, HRR genes are associated with increased risk of breast, ovarian and pancreatic cancer (*BRCA1*, *BRCA2*, *ATM*, *BRIP1*, *PALB2*, *BARD1*, *CHEK2*, *RAD51C* and *RAD51D).* Alterations in some of the DDR genes are associated with cancer predisposition syndromes (e.g. *BRCA1*, *BRCA2*, *ATM*, *FANCL*, *PALB2*, *RAD51C*, *BRIP1*, FA genes, MMR genes) (64, 100, 101, 110, 115, 116, 118–127). Germline pathogenic mutations in MMR genes (*MLH1, MSH2, MSH6* and *PMS2*) are the key cause of Lynch syndrome (LS) – an inherited cancer predisposition syndrome leading to increased risk of particularly colorectal cancer and other LS-associated cancers. Patients with LS have a twofold elevated risk of incidence of prostate cancer compared with the general population (128).

### Prognosis for HRRm prostate cancer

Among HRR alterations, germline pathogenic *BRCA* mutations in particular are associated with a more aggressive prostate cancer and worse outcomes for patients with localised prostate cancer and mCRPC compared to non-carriers. The presence of a germline pathogenic *BRCA* mutation in prostate cancer is associated with a more aggressive phenotype, such as higher Gleason scores, nodal involvement and presence of metastases at diagnosis, as shown in large retrospective analyses (93, 94). *BRCA2* was reported to be an independent prognostic factor in a multivariate analysis, where patients with germline pathogenic *BRCA* mutations had overall worse outcomes than non-BRCAm patients in a cohort with localised disease as well as in the overall cohort (93, 94). Another study reported that the combined rate of germline *BRCA/ATM* alterations was significantly higher in patients with lethal prostate cancer than in patients with localised prostate cancer, and patients with germline *BRCA/ATM* alterations with either localised disease or a diagnosis with metastases, had a shorter prostate-cancer-specific survival compared with non-carriers (129).

Once prostate cancer becomes castration-resistant and progresses to a metastatic stage (mCRPC) the disease is not curable and treatment must focus on extending life, delaying disease progression and improving quality of life (130, 131). Germline mutations in HRRm have been found in around 12% of patients with mCRPC (78). There has been conflicting evidence reported for the association of HRRm and prognosis for patients with mCRPC. Annala et al. reported worse outcomes for germline DDR carriers (17/22 were *gBRCAm*) compared with non-carriers when treated with first-line androgen receptor signaling inhibitors (132). However, more recently Antonarakis et al. reported that patients with *BRCA/ATM* mutations (n=9) do better on first-line NHA than those without mutations in these genes (133). These findings are based on a relatively small number of patients with HRRm. A retrospective analysis showed similar OS outcomes for patients with metastatic prostate cancer and with germline mutations in DDR genes compared with those without; however, somatic alterations in DDR genes were not assessed, and a significant proportion of patients were treated with PARPi or platinum-based chemotherapy, which might have contributed to better outcomes for patients with DDRm (50, 96). A prospective study, PRO-REPAIR, evaluated prevalence of germline DDR mutations and their impact on outcomes for patients with mCRPC (99). This trial enrolled unselected patients with mCRPC and screened for germline alterations in 107 DDR genes with the primary objective to assess the impact of germline alterations in *ATM/BRCA1/BRCA2/PALB2* on cause-specific survival (CSS) from diagnosis of mCRPC. In PRO-REPAIR, 16% of patients had a germline mutation in a HRR gene (most commonly *BRCA* and *ATM* alterations). Although numerically the CSS was shorter in patients with *ATM/BRCA1/BRCA2*, the difference was not statistically significant; however, patients with germline *BRCA2* mutation had considerably shorter CSS than non-carriers and *BRCA2* was an independent prognostic factor, where sequence and type of treatment may impact the outcomes (99). Castro et al. reported that treatment sequence is important for prognosis, with patients with mCRPC and DDR mutations having worse outcomes overall but better outcomes following first-line NHA treatment (99). These observations might explain conflicting observations in different studies. In addition to alterations in HRR genes, genomic instability might also be associated with worse outcomes, such as with risk of biochemical recurrence and metastases (134, 135).

### Treatment options for patients with DDRm prostate cancer

High unmet clinical need and poor prognosis for patients with prostate cancer and DDR has triggered an active development of targeted treatment options for these patients (136). As described above, based on the principle of synthetic lethality, cells deficient in the HRR pathway are sensitive to PARP inhibitors (137, 138) (**Figure 1**). PARP inhibitors as monotherapy have demonstrated efficacy in patients with evidence of deficiency in the HRR pathway in the tumour and have been approved for treatment of HRRm mCRPC (136). The clinical benefit of PARPi in combination with NHA has been demonstrated in biomarker-selected and biomarker-unselected populations in first-line mCRPC (**Table 1**) (36–39), with approval in some regions, providing additional efficacious treatment options for these patients.

Platinum-based chemotherapy also leads to DNA damage, which is repaired by HRR pathway. Increased platinum sensitivity in tumours deficient in HRR pathway (ie BRCAm) has been reported in other tumour types, such as breast or ovarian cancer (139, 140) Retrospective analyses indicated encouraging anti-tumour activity of platinum-based chemotherapy in advanced prostate cancer patients with BRCA alterations or some DDR alterations with higher PSA response rates in patients with DDR/BRCA alterations compared to patients without (141–143), although these findings need to be validated in a prospective setting.

*ATM*-deficient cells are dependent on ATR activity, which leads to sensitivity to ATR inhibition preclinically; clinical trials with ATR inhibitors as monotherapy or in combinations are ongoing, including in biomarker-selected patients with ATM- or DDR-deficiency in advanced solid tumours, including prostate cancer, as reviewed recently by Ngoi et al. (144).

Anti-PD1 antibody (Pembrolizumab) is approved by the Food and Drug Administration (FDA) to treat cancers with MMR mutations or MSI-H, including prostate cancer (145, 146). *CDK12* inactivation results in tandem duplications in the genome leading to increased fusions and mutations and might lead to increased antigens. Clinical trials with immune checkpoint inhibitors are ongoing for patients with *CDK12*m and advanced prostate cancer (147–149).

## Conclusions/takeaway

Prostate cancer is the third most common cancer worldwide, and metastatic prostate cancer is associated with poor outcomes and high mortality. There are various genomic alterations commonly associated with prostate cancer, and alterations in the HRR pathway of DDR are prevalent in prostate cancer, ranging from 23–28% (25, 42, 43, 46, 78). Germline alterations in several HRR genes, such as *BRCA, ATM* and others, are associated with increased prostate cancer risk, and are generally associated with worse prognosis for patients with prostate cancer. Alterations in DDR genes in tumours tend to be an early event in prostate tumourigenesis and are associated with more aggressive disease and likelihood of metastasis (97). With current breakthroughs regarding targeted treatments, PARP inhibitors as monotherapy are an option for patients with HRR alterations in mCRPC, who have progressed on NHA (136) and in some countries, are available in combination with NHA in a broad population of 1L mCRPC (36, 55, 99). Clinical trials are ongoing that are evaluating ATR inhibitors in all solid tumours, including prostate cancer, as reviewed by Ngoi et al. (144). Given the association of some DDR genes with worse prognosis, and DDR alterations being an early event in prostate cancer, future clinical trials for patients with DDRm and an earlier stage of disease are important to improve outcomes for these patients.

## Author contributions

NL and AB developed the initial draft of the manuscript. NL, AB, EH, CA and JCB made substantial contributions to the concept and content of review. JA, JK and CA contributed to writing parts of the manuscript. All authors contributed to the article and approved the submitted version.

## Footnotes

2 Although PPP2R2A was originally included in 15 HRR genes, no benefit of olaparib over control therapy was noted in patients with alterations in PPP2R2A in the PROfound trial (84)

## Glossary

**Table:**

ADT	androgen deprivation therapy
BER	base excision repair
BRCAm	mutations in *BRCA1* and *BRCA2*
CI	confidence interval
CRPC	castration-resistant prostate cancer
CSS	cause-specific survival
DDR	DNA damage response
FA	Fanconi anaemia
gLOH	genome-wide loss of heterozygosity
HRD	homologous recombination deficiency
HRR	homologous recombination repair
LS	Lynch syndrome
mCRPC	metastatic castration-resistant prostate cancer
mCSPC	metastatic castration-sensitive prostate cancer
MMR	mismatch repair
MSI-H	miscrosatellite instability-high
NCCN	National Comprehensive Cancer Network
NHA	new hormonal agent
OR	odds ratio
OS	overall survival
PARP	Poly(ADP-ribose) polymerase
PARPi	Poly(ADP-ribose) polymerase inhibitor
PSA	prostate-specific antigen
rPFS	radiographic progression-free survival
RR	relative risk
SRR	standardised relative risk
SSBs	single-strand breaks.
